# Assessment of Rhinoplasty Outcomes with FACE-Q Rhinoplasty Module: Norwegian Linguistic Validation and Clinical Application in 243 Patients

**DOI:** 10.1097/GOX.0000000000002448

**Published:** 2019-09-30

**Authors:** Amin Kalaaji, Stine Dreyer, Jakob Schnegg, Lena Sanosyan, Tatjana Radovic, Ivana Maric

**Affiliations:** From the Oslo Plastikkirurgi Clinic, Oslo, Norway.

## Abstract

Patient satisfaction after rhinoplasty is a growing area of research. The FACE-Q Rhinoplasty Module, used to assess these values, requires translation to national languages.

**Methods:**

Fourteen questions assessing the Satisfaction with Nose Scale and Adverse Effects Checklist of FACE-Q Rhinoplasty Module were translated to Norwegian with adherence to the Mapi Research Trust guidelines. Answers were processed by QuestBack anonymously. Of the 243 patients undergoing rhinoplasty at Oslo Plastic Surgery Clinic, 214 patients were reachable by e-mail.

**Results:**

Response rates to the pre- and postoperative questionnaire were 23% and 32%, respectively. Responses for somewhat or very satisfied with the nose (pre- versus postoperative) were: overall size of the nose (16.3% versus 61.7%); how straight the nose looks (22.4% versus. 58.3%); how well the nose suits the face (12.2% versus 60%); length of the nose (20.4% versus 68.4%); width of the nose at the bottom (26.6% versus 55%); bridge of the nose (14.3% versus 55%); how the nose looks in photographs (10.2% versus 50%), and tip of the nose (16.3% versus 48.3%). Adverse effects (pre- versus postoperative) were moderate or extreme difficulty breathing through the nose (28.6% versus 35%); tenderness (6.1% versus 23.7%); skin of the nose looking thick or swollen (14.6% versus 30.5%); and unnatural bumps or hollows on the nose (55.1% versus 53.3%).

**Conclusions:**

Satisfaction levels in rhinoplasty patients are not as high as in other cosmetic surgery procedures, such as breast augmentation. However, compared with baseline, satisfaction levels showed great improvement postoperatively. The Rhinoplasty Module seems useful in evaluating outcome of rhinoplasty. We encourage application of this clinical outcome of rhinoplasty in and among centers.

## INTRODUCTION

The nose is a distinctive facial feature of immense aesthetic importance to the identity of every human being.^1^ To achieve optimal facial beauty, the nose must fit the face harmoniously and inconspicuously. However, the nose is not just a facial beauty feature, but a critical sensory organ vital to one of the essential functions of life: breathing.^2^ Rhinoplasty is one of the most complex procedures in plastic surgery.^3–5^ It is also one of the earliest known surgeries performed to increase a patient’s quality of life (QoL) after a disease-or trauma-related deformity of the nose by restoring functional and aesthetic capacities.^6^ Despite a 2% decrease in the number of rhinoplasties performed, this procedure still remains one of the most popular cosmetic plastic surgeries. In 2017, more than 218,000 nose-reshaping operations were performed in the United States.^7^

The ultimate goal of aesthetic rhinoplasty is to create a harmonious and natural-appearing nose that assimilates into the surrounding face with no visible sign of previous intervention and that allows a patient to breathe comfortably without restriction.

Postoperative complications and mortality rates have traditionally served as mainstay in clinical outcome research.^8^ Though the importance of these measures remains, evaluation of patient satisfaction and QoL are becoming increasingly relevant in cosmetic and reconstructive surgery.^8–13^ Comprehensive assessment of surgical outcome demands rigorously developed patient questionnaires that have sufficient reliability, validity, and responsiveness,^8^ as these would facilitate the comparison of techniques, the quantification of positive outcomes, and allow for identification of individuals that most likely benefit from the respective surgery.^11,12^ As implied by Pusic et al,^8^ data yielded from patient questionnaires that are not psychometrically tested (“ad hoc questionnaires”) may not be viable for making confident conclusions on the impact and effectiveness of plastic surgery due to lacking reliability and validity.

Many studies are performed to evaluate the effects of rhinoplasty on the patients’ life^14^, for which several questionnaires are used (e.g., Rhinoplasty Outcome Evaluation;^15^ Sino-Nasal Outcome Test;^16^ Nasal Obstruction and Septoplasty Effectiveness Scale^17^). However, information on structural validity and internal consistency are lacking for many of these instruments,^18^ and none includes a wide variety of questions to evaluate separate parts of the nose and how this feature reflects QoL before and after surgery.

The FACE-Q rhinoplasty module^19^ is an instrument designed to evaluate patient-reported outcomes (PRO) before and after undergoing rhinoplasty and to assess adverse effects regarding the nose. This module requires translation to national languages.^20^

The present study was conducted to evaluate patient satisfaction with the outer appearance of the nose, its function, and changes. The first of our 2 main goals was to apply an internationally validated tool for rhinoplasty to evaluate the outcomes of the procedure. This article is a detailed guide for rhinosurgeons who want to validate the FACE-Q rhinoplasty module in their country, including the whole translation process: stages and nuances, validation process, and study methods. By using this tool in daily practice, surgeons are able to collect fully anonymous responses from patients and build solid evidence-based data regarding their methods, techniques, and strategies. The second goal was to measure QoL of patients in relation to rhinoplasty. Patients were given an opportunity to rate how their satisfaction with the nasal appearance has changed after surgery. Moreover, we believe that common adverse events of rhinoplasty are also an important aspect to be considered when discussing changes in QoL. The anonymous style of the evaluation ensured honesty and objectivity from the patients when answering the questions. In light of the fact that many publications reporting technical details often do not include satisfaction of PRO conducted anonymously, we hope this work will contribute new knowledge to the rhinoplasty field.

## METHODS

### Patients

A total of 243 patients who underwent rhinoplasty at the Oslo Plastic Surgery Clinic between 2007 and 2016 had a valid e-mail address and were contacted to enroll in the study. Patients who were followed up <1 year and those not reachable by e-mail were excluded from the study.

### Questionnaire

The FACE-Q rhinoplasty module is a part of the FACE-Q scales and is owned by Memorial Sloan Kettering Cancer Center (New York, N.Y.), which holds the copyright of the original and all translated versions of the questionnaire. It consists of 14 validated questions that were divided into 2 separate matrixes to measure PRO: Satisfaction with Nose (10 items) and Adverse Effects Regarding the Nose (4 items). (Table 1–2)

**Table 1.** Satisfaction with Nose

	Very Dissatisfied	Somewhat Dissatisfied	Somewhat Satisfied	Very Satisfied
a. The overall size of your nose?	1	2	3	4
b. How straight your nose looked?	1	2	3	4
c. How well your nose suited your face?	1	2	3	4
d. The length of your nose?	1	2	3	4
e. The width of your nose at the bottom (from nostril to nostril)?	1	2	3	4
f. How the bridge of your nose looked (where glasses sit)?	1	2	3	4
g. How the tip of your nose looked?	1	2	3	4
h. The shape of your nose in profile (side view)?	1	2	3	4
i. How your nose looked in photos?	1	2	3	4
j. How your nose looked from every angle?	1	2	3	4

These questions ask about how you look right now. For each question, circle only one answer. With your nose in mind, in the past week, how satisfied or dissatisfied have you been with.

**Table 2.** Adverse Effects on the Nose

	Not at All	A Little	Moderately	Extremely
a. Difficulty breathing through your nose?	1	2	3	4
b. Tenderness (e.g. when wearing sunglasses)?	1	2	3	4
c. The skin of your nose looking thick or swollen?	1	2	3	4
d. Unnatural appearing bumps or hollows on your nose?	1	2	3	4

These questions ask about problems you may be currently experiencing. With your nose in mind, in the past week, how much have you been bothered by.

The first group of questions (*Satisfaction with Nose*) concern patient satisfaction with the current appearance of the nose and the week before completing the questionnaire. For each question, patients were asked to rate their level of satisfaction/dissatisfaction on a scale of 1 to 4 (1 being “very dissatisfied”; 2, “somewhat dissatisfied”; 3, “somewhat satisfied”; 4, “very satisfied”) (Table 1). The second group of questions (*Adverse Effects Regarding the Nose*) deals with problems patients might be experiencing the preceding week. Four answer options were provided (1, “not at all”; 2, “a little”; 3, “moderately”; 4, “extremely”) (Table 2).

### Translation Process

After permission was obtained from the development team, the FACE-Q rhinoplasty module was translated into Norwegian using a multistep process. The internationally accepted translation methodology recommended by Mapi Research Trust (Lyon, France)^21^ was strictly followed during all stages.

**Fig. 1.**
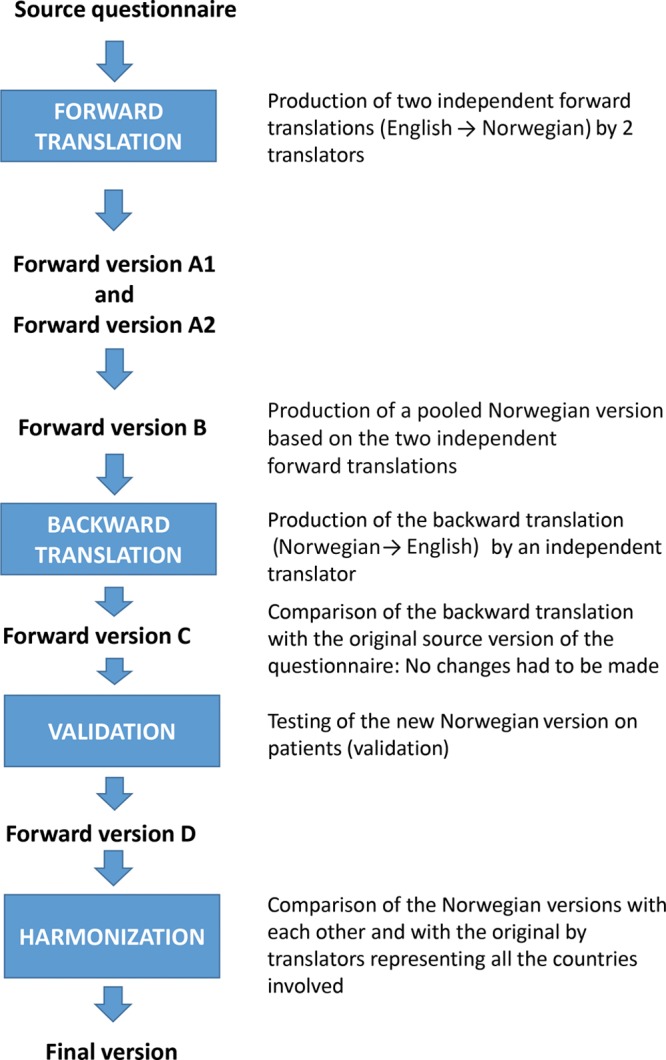
Translation process. In Phase 1 (forward translation), 2 independent forward translations (English to Norwegian) were produced (Forward version A1 and Forward version A2). A pooled Norwegian version (Forward version B) was then produced based on the 2 independent forward translations. Phase 2 (backward translation) was performed from Norwegian to English. Comparison between the backward version and the original source questionnaire yielded Forward version C. In Phase 3 (validation), Forward version C was tested (validated) on a patient sample, obtaining Forward version D. In a final step (harmonization), forward versions were compared with each other and the original source questionnaire and matched to ensure conceptual equivalence. After final proof reading, the final version of the questionnaire in Norwegian was obtained. Adapted with permission from *Health Qual Life Outcomes*. 2003;1:70.^22^

Phase 1 was the forward translation step for which 2 local professional translators, both American and Norwegian, with previous experience in English-Norwegian translation work, and a local project manager were recruited. In this step, each translator independently produced a forward translation (English to Norwegian), yielding forward versions A1 and A2. Based on the 2 forward translations, 1 pooled Norwegian version (forward version B) was created. Both translators and the local project manager were involved in this process, and no interpretation problems were experienced. Phase 2 was the backward translation step in which an independent translator interpreted and retranslated the pooled Norwegian version (forward version B) into the language of origin. The newly obtained English version was then compared with the source questionnaire by the “backward translator” and the local project manager. These matched perfectly and, therefore, no adaptations of the first Norwegian version were necessary (forward version C). Phase 3 was intended for validation of the Norwegian version and involved 8 patients who were native speakers of the target language, obtaining forward version D. After harmonization of the target language versions with each other and with the original by professional translators representing all the countries involved and final proofreading, the final version was obtained. A quality control step by the Mapi Research Trust was performed during all stages (Fig. 1).

### Measures to Ensure Anonymity

A third, independent party, QuestBack return mail system (QuestBack AS, Oslo, Norway), was used to automatically process survey answers anonymously and return the results in form of diagrams and figures to the authors. The e-mail addresses of all patients were registered in the clinic records, enabling us to send the questionnaire via e-mail.

This study did not require approval by an institutional review board because it was conducted anonymously. Patients gave their consent to use their answers anonymously.

The authors followed the guiding principles from the Declaration of Helsinki.

## RESULTS

Of the total 243 patients (210 females and 33 males) undergoing rhinoplasty at our clinic, 214 were reachable via e-mail. The mean age of patients was 28.5 years (range 17–68). Mean follow-up time was 4.6 years (range 1–12). The preoperative questionnaire was completed by 49 of 214 patients (response rate: 23%) and the postoperative questionnaire by 60 of 185 patients (response rate: 32%).

### Satisfaction with Nose

Before the operation, few patients were “somewhat” or “very satisfied” with “the overall size of the nose” (16.3%); “how straight the nose looked” (22.4%); “how well the nose suited the face” (12.2%); “the length of the nose” (20.4%); “the width of the nose at the bottom” (from nostril to nostril) (26.6%); “how the bridge of the nose looked” (where eyeglasses sit) (14.3%); “how the tip of the nose looked” (16.3%); “the shape of the nose in profile” (side view) (8.2%); “how the nose looked in photographs” (10.2%); and “how the nose looked from every angle” (6.3%). (Table 3)

**Table 3.** Satisfaction with Nose Scale

To What Degree Are You Satisfied With	Very Dissatisfied n (%)		Somewhat Dissatisfied n (%)		Somewhat Satisfied n (%)		Very Satisfied n (%)	
	Preoperatively	Postoperatively	Preoperatively	Postoperatively	Preoperatively	Postoperatively	Preoperatively	Postoperatively
Overall size of the nose	31 (63.3)	11 (18.3)	10 (20.4)	12 (20.0)	5 (10.2)	22 (36.7)	3 (6.1)	15 (25.0)
How straight the nose looks	22 (44.9)	16 (26.7)	16 (32.7)	9 (15.0)	5 (10.2)	23 (38.3)	6 (12.2)	12 (20.0)
How well the nose suits the face	28 (57.1)	7 (11.7)	15 (30.6)	17 (28.3)	5 (10.2)	19 (31.7)	1 (2.0)	17 (28.3)
Length of the nose	23 (46.9)	7 (11.7)	16 (32.7)	12 (20.0)	6 (12.2)	19 (31.7)	4 (8.2)	22 (36.7)
Width of the nose at the bottom (from nostril to nostril)	20 (40.8)	13 (21.7)	16 (32.7)	14 (23.3)	9 (18.4)	19 (31.7)	4 (8.2)	14 (23.3)
Bridge of the nose (where eyeglasses sit)	27 (55.1)	15 (25.0)	15 (30.6)	12 (20.0)	5 (10.2)	22 (36.7)	2 (4.1)	11 (18.3)
Tip of the nose	34 (69.4)	16 (26.7)	7 (14.3)	15 (25.0)	5 (10.2)	17 (28.3)	3 (6.1)	12 (20.0)
Shape of the nose in profile (side view)	39 (79.6)	13 (21.7)	6 (12.2)	13 (21.7)	2 (4.1)	19 (31.7)	2 (4.1)	15 (25.0)
How the nose looks in photos	37 (75.5)	16 (26.7)	7 (14.3)	14 (23.3)	4 (8.2)	21 (35.0)	1 (2.0)	9 (15.0)
How the nose looks from every angle	32 (64.6)	14 (23.3)	14 (29.2)	19 (31.7)	3 (6.3)	21 (35.0)	0 (0.0)	6 (10.0)

Before undergoing surgery, patient dissatisfaction (“very dissatisfied”) was particularly high in the outcomes measure “the shape of the nose in the profile/side view” (79.6%), followed by “how the nose looks in photographs” (75.5) and “how the tip of the nose looks” (69.4%). No patient was “very satisfied” with “how the nose looked from every angle.”

Postoperatively, considerably more patients were “somewhat” or “very satisfied” with all Satisfaction with Nose items. (Figs. 3–12) Postoperative ratings are summarized in Table 3.

**Fig. 2.**
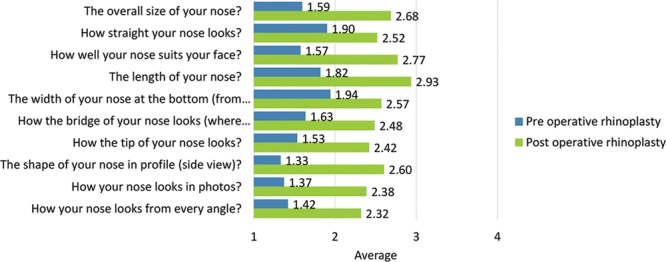
Total scale scores pre- and postrhinoplasty: Satisfaction with nose (10 items).

**Fig. 3.**
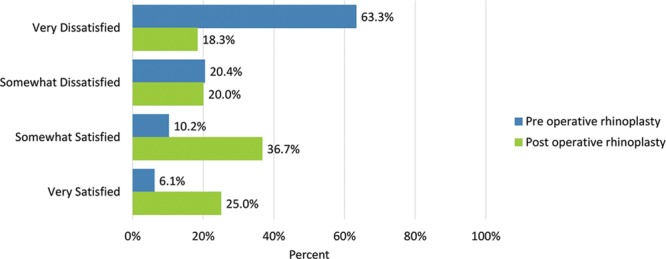
Satisfaction or dissatisfaction with the overall size of the nose.

**Fig. 4.**
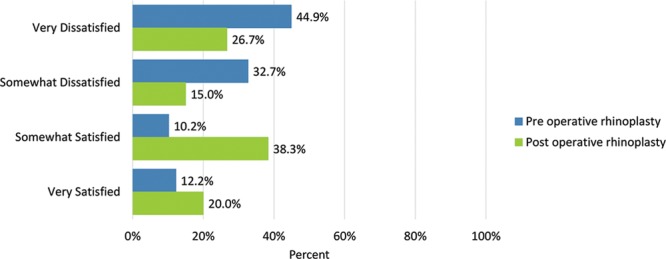
Satisfaction or dissatisfaction with how straight the nose looked.

**Fig. 5.**
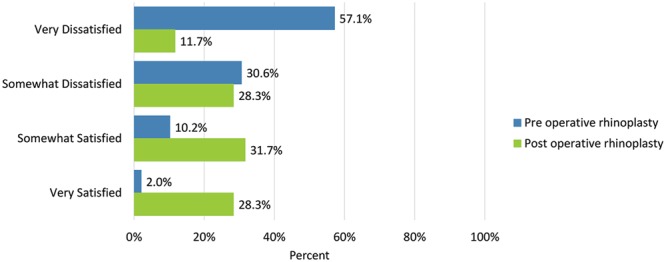
Satisfaction or dissatisfaction with how well the nose suited the face.

**Fig. 6.**
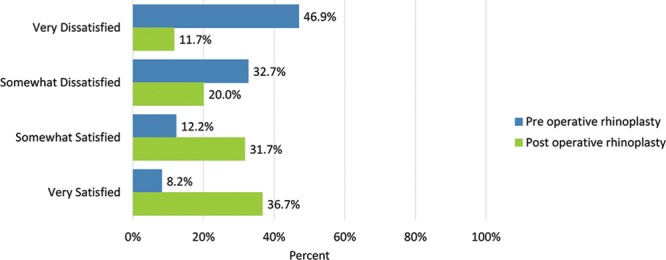
Satisfaction or dissatisfaction with the length of the nose.

**Fig. 7.**
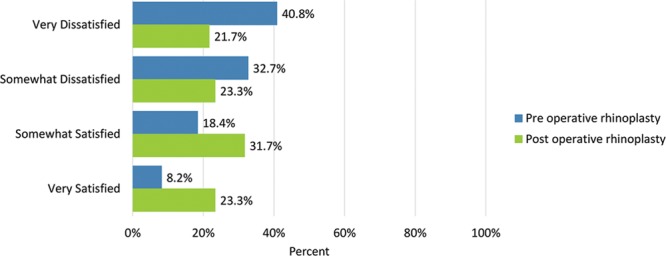
Satisfaction or dissatisfaction with the width of the nose at the bottom (from nostril to nostril).

**Fig. 8.**
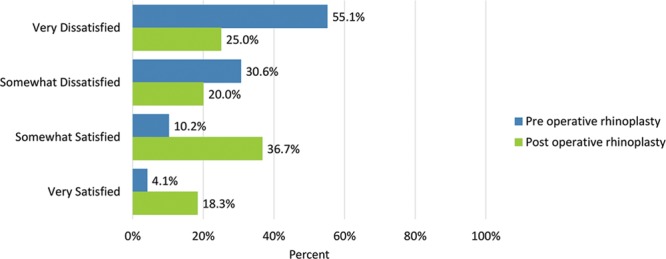
Satisfaction or dissatisfaction with how the bridge of the nose looked (where eyeglasses sit).

**Fig. 9.**
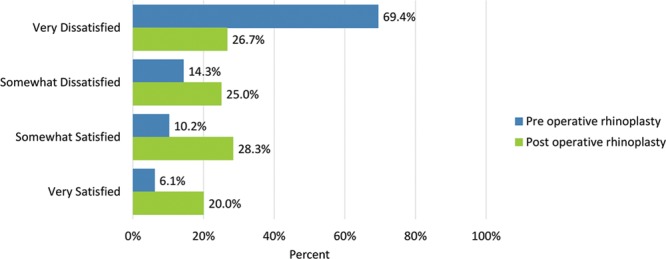
Satisfaction or dissatisfaction with how the tip of the nose looked.

**Fig. 10.**
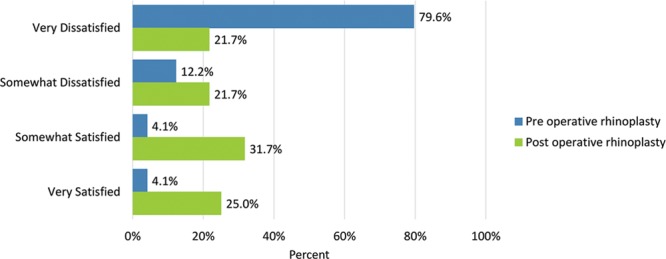
Satisfaction or dissatisfaction with the shape of the nose in profile (side view).

**Fig. 11.**
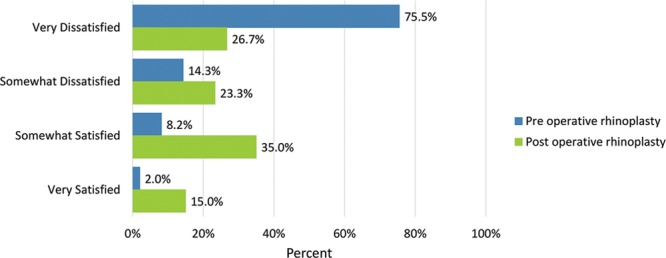
Satisfaction or dissatisfaction with how the nose looked in photos.

**Fig. 12.**
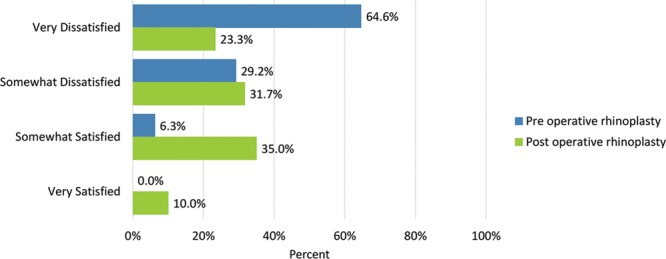
Satisfaction or dissatisfaction with how the nose looked from every angle.

**Fig. 13.**
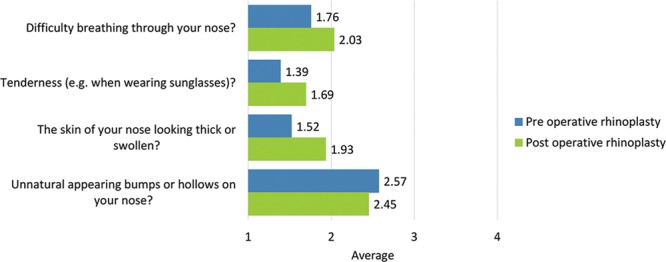
Total scale scores pre- and postrhinoplasty: adverse effects regarding the nose (4 items).

Total scale scores pre- and postrhinoplasty are provided in Figure 2.

### Adverse Effects regarding the Nose

Preoperative adverse effects regarding the nose were moderate or extreme “difficulty breathing through the nose” (28.6%), “tenderness such as when wearing sunglasses” (6.1%), “the skin of the nose looking thick or swollen” (14.6%), and “unnatural bumps or hollows on the nose” (55.1%).

**Fig. 14.**
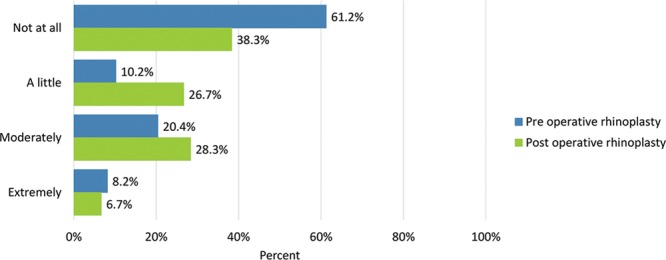
Bothersomeness of difficulty breathing through your nose.

**Fig. 15.**
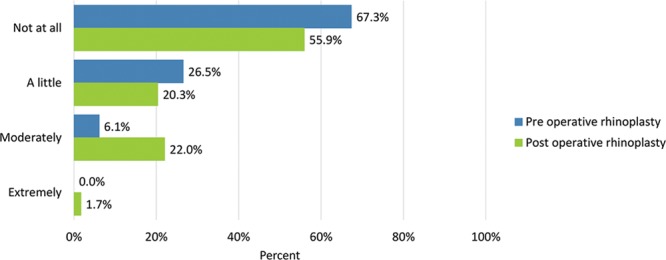
Bothersomeness of tenderness (eg, when wearing sunglasses).

**Fig. 16.**
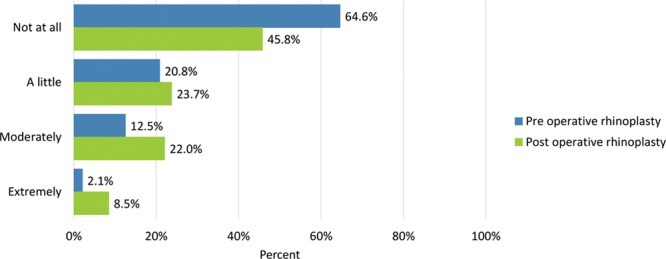
Bothersomeness of the skin of the nose looking thick or swollen.

**Fig. 17.**
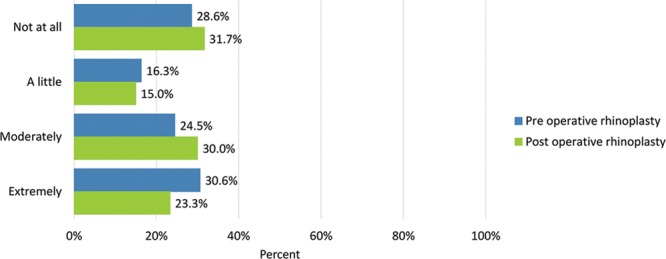
Bothersomeness of unnatural bumps or hollows on the nose.

Adverse effects were generally rated more severe after undergoing surgery: moderate or extreme “difficulty breathing through the nose” (35%), “tenderness such as when wearing sunglasses” (23.7%), “the skin of the nose looking thick or swollen” (30.5%), and “unnatural bumps or hollows on the nose” (53.3%) (Figs. 14–17). Fewer patients cited extreme difficulty breathing through the nose and unnatural bumps or hollows on the nose after the operation (Table 4).

**Table 4.** Adverse Effects Checklist

How much have you been bothered by the following?	Not at All n (%)		A Little n (%)		Moderately n (%)		Extremely n (%)	
	Preop	Postop	Preop	Postop	Preop	Postop	Preop	Postop
Difficulty breathing through the nose	30 (61.2)	23 (38.3)	5 (10.2)	16 (26.7)	10 (20.4)	17 (28.3)	4 (8.2)	4 (6.7)
Tenderness (eg, when wearing sunglasses)	33 (67.3)	34 (55.9)	13 (26.5)	12 (20.3)	3 (6.1)	13 (22.0)	0 (0.0)	1 (1.7)
The skin of the nose looked thick or swollen	32 (64.6)	28 (45.8)	10 (20.8)	14 (23.7)	6 (12.5)	13 (22.0)	1 (2.1)	5 (8.5)
Unnatural bumps or hollows of the nose	14 (28.6)	19 (31.7)	8 (16.3)	9 (15.0)	12 (24.5)	18 (30.0)	15 (30.6)	14 (23.3)

Before/after the operation, how much have you been bothered by the following?

Total scale scores pre- and postrhinoplasty are provided in Figure 13.

## DISCUSSION

### Importance of Patient-Reported Outcomes Measures

In aesthetic outcome evaluation, the tide is turning from objective and physician-reported outcomes measures, such as comparison of pre- and postoperative photographs, toward patient-reported outcomes measures (PROMs).^11,23^ Exempt of interpretation by clinicians, these instruments provide insight into subjective perspectives of the patients in relation to satisfaction, QoL, and adverse effects after a procedure.^24^ PROMs enable patients to directly express their level of satisfaction and also aid physicians in the intricate process of clinical decision-making to improve outcomes.^25–27^ This is of particular interest in the field of rhinosurgery, in which understanding of the expectations and concerns of patients is especially important to accomplish satisfactory results.^3,28,29^

Rhinoplasty undoubtedly represents one of the most difficult cosmetic surgeries.^3–5^ Quantification of PRO satisfaction would enable the surgeon to assess the success for each technique from a patient perspective and determine the technique with the best outcome.^3,24^

Evaluation of outcomes from the patient’s point of view is relevant to cosmetic surgery, given that many essential items such as appearance and function are evaluated best through self-assessment by the patient.^11,19^ Ultimately, it is the patient who has to be happy with the result.^30^

By application of PROs in the clinical practice, physicians are obliged to be meticulous in patient consultation, that is, assessing patient expectations, considering their feasibility, and explaining the realistic results to patients. Furthermore, constant patient feedback about the satisfaction with the postoperative results and adverse effects following surgery allows surgeons to analyze strengths and weaknesses of their surgical approach and may contribute to technique advancement, for example, by efforts to reduce trauma.

### The FACE-Q Rhinoplasty Module

The FACE-Q rhinoplasty Module is a rigorously developed PRO instrument to evaluate QoL and that complies with the requirements of international guidelines.^11,19^ This tool is designed to specifically evaluate cosmetic and psychosocial aspects associated with rhinoplasty, and it has been suggested to be the most suitable instrument to evaluate aesthetic outcome.^18^

### Translation Process

In the translation process, prevention of loss of information, validity, reliability are of utmost importance.^31^ To yield an appropriate Norwegian version of the FACE-Q rhinoplasty module, steps were followed strictly in accordance with international translation recommendations by Mapi Research Trust^21^ (Fig.1). Other authors used a similar approach to translate this module into national languages.^20,32^

### Comparison with Other Studies

Schwitzer et al^3^ were the first to use the FACE-Q to measure changes in patient satisfaction with facial appearance overall, appearance of the nose, and the changes in QoL in the rhinoplasty population. The authors demonstrated significant improvement of pre- and postrhinoplasty scores in facial appearance, social function, and psychological well-being. Moreover, improved patient satisfaction could be illustrated with the nasal appearance for all items, including size, shape, profile, in the mirror, and in photographs.^3^

The creators of the FACE-Q rhinoplasty module^19^ reported an increased score on the Satisfaction with Nose and Nostrils scale when they compared 23 patients pre- and postoperatively.

Radulesco et al^29^ indicated that the items “how the nose looks from every angle,” “how the bridge of the nose looks (where eyeglasses sit),” “how well the nose suits the face,” and “how the nose looks in photos” showed the best discriminating power between rhinoplasty patients and the control group. In accordance, these items, together with “shape of the nose in profile (side view),” showed the highest rates of preoperative dissatisfaction in the patients of the current study. The authors concluded that these items might best reflect the general appearance of the nose, combining all dysmorphoses and problems in relation to the dorsal hump, a frequent prominent feature of the nose.^29^

Klassen et al^19^ demonstrated that “the skin of your nose looking thick or swollen” was the most commonly experienced adverse effect after rhinoplasty; more than half the patients were affected. This concurs with data from the current study: 23.7% of patients were affected mildly; 22%, moderately; and 8.5%, extremely by a thick/swollen appearance of the skin of the postoperative nose. This finding is hardly surprising, given that skin swelling is considered normal sequela after surgery.^30,33^

In keeping with previous findings,^19^ slightly >40% of patients were bothered by postoperative tenderness (eg, when wearing sunglasses) in the present study. Klassen et al^19^ revealed that this adverse effect was associated with time since surgery. More than 60% of the patients reported a little, moderate, or extreme difficulty breathing through the nose postoperatively. A possible explanation for this finding might be that transient edema is a normal effect after surgery that causes nasal airway obstruction over a certain period. Notably, the number of respondents who reported extreme difficulty breathing through the nose preoperatively decreased even slightly after surgery. This might be attributable to the fact that the underlying cause in these patients, such as septal deviation, was corrected through surgery.

We found it important to assess preexisting adverse effects to illustrate that many of the mentioned items had been present before patients underwent surgery. Some of the items assessed in the FACE-Q adverse events checklist stem from the same underlying cause. For example, difficulty breathing and swollen/thick skin might be caused collectively by postoperative edema. Patients tend to report the more frequent (often transient) adverse effects that are seen as inevitable sequelae of the operation rather than as severe complications.^33^

Fleury et al^33^ pointed out that many more possible complications after rhinoplasty, such as epistaxis, septal hematoma, infection, telangiectasia, and skin necrosis, are documented in the literature. However, many of these physician-reported effects are seldom and rarely experienced by the individual patient. It is conceivable that information about adverse events stemming from experience reports of other patients might be more relevant to the patient. This becomes evident from the abundant online communities that have blossomed in popularity over the past decade, in which members report their plastic surgery experience to others. Therefore, preoperative patient consultation should not only involve data from physician-reported adverse events but also from PRO, to deepen patient understanding of what to expect most realistically from the procedure.^33^

### Strengths

Few studies in rhinoplasty follow-up with patients scientifically and anonymously. However, it is important for evaluation of different techniques and noting the levels of satisfaction among patients. This might enable rhinosurgeons to learn more about patient needs and expectations, thereby reducing complaints and complications.

Assessment of preoperative values for satisfaction with the nasal appearance and preexisting adverse effects enables surgeons to identify patients who will potentially be less satisfied with the surgical outcome,^30^ as individuals who have very low^34^ or higher/above normal scores are predicted to be less satisfied after surgery.^29^ Another strength of the current study lies in its anonymous design, which reduces bias and makes data more reliable. Patients do not feel coerced into stressing preoperative adverse events regarding the nose to warrant surgery.

Furthermore, all patients were operated on by the same surgeon (A.K.) in a single practice, allowing for good comparability between results. Last, the translation process was conducted with strict adherence to the international guidelines and therefore brings forth a ready tool to be used by other surgeons in Norway and can serve as guidelines for other colleagues.

### Limitations

The following limitations were encountered in the study. The response rate of patients in the study was relatively low. Patients who are less satisfied with the outcome are potentially more willing to respond to the questionnaire to diminish scores and assure their grievance about the outcome.This might bias the results toward a poorer outcome.

Inherent in the retrospective design of the current study, most questionnaires (including those assessing the preoperative status) were completed postoperatively, whereas few were completed before the surgery. Ideally, the surveys are provided preoperatively and at consistent times postoperatively to provide the most reliable data. Operations were performed over a considerable period, which makes outcomes less comparable. Changes in satisfaction with the nasal appearance may fluctuate over time. A long time after surgery, respondents might rate the outcome less favorably than earlier in the postsurgical period, when the improvement to the preoperative state was more evident. Furthermore, the presence of adverse effects was assessed only at 1 specific time point which varied considerably among patients. Longer after the surgery, adverse effects might be less present than nearer the time of the operation. Evaluation of adverse events at multiple time points and synchronized between patients would thus be useful to reveal potential changes. Last, the Satisfaction with Nostrils questionnaire was not applied in the current study because this scale might not be suitable for all patients wishing to undergo rhinoplasty.^29^

## CONCLUSIONS

The FACE-Q rhinoplasty module was translated into Norwegian by strict adherence to the translation method used by Mapi Research Trust and the results of its application in our daily practice are presented.

Our findings indicate that the FACE-Q rhinoplasty module has great potential in measuring pre- and postoperative QoL in patients electing to undergo rhinoplasty. Provisional results of patient satisfaction levels after aesthetic rhinoplasty seem modest compared with the reported outcomes of other cosmetic procedures, such as breast implantation and augmentation mastopexy. This implies that preoperative counseling must allude to the relatively high percentage of patients who are not fully satisfied. Nevertheless, compared with baseline, substantial improvement in satisfaction could be noted.

Additional data from rhinoplasty patients and the normal population in the form of multicenter studies will be necessary to further delineate the importance of this outcomes instrument and to conclude with greater certainty. Therefore, we encourage our colleagues from all over the world to conduct rhinoplasty studies based on PROMs.

## ACKNOWLEDGMENT

The authors thank Cathrine W. Knudsen, MD, PhD, for her contribution to the translation process.
