# Assessment of Rhinoplasty Outcomes with FACE-Q Rhinoplasty Module: Norwegian Linguistic Validation and Clinical Application in 243 Patients: Erratum

**DOI:** 10.1097/GOX.0000000000002773

**Published:** 2020-03-25

The authors of the September 2019 article titled “Assessment of Rhinoplasty Outcomes with FACE-Q Rhinoplasty Module: Norwegian Linguistic Validation and Clinical Application in 243 Patients,” published in *PRS Global Open* on 30 September 2019, wish to make the following clarification:

The FACE-Q is a trademarked patient reported outcome instrument. The “Satisfaction with Nose” and “Adverse Effects Checklist” described and reproduced in this article are similarly trademarked; the copyright is retained by the Q-Portfolio. If readers would like to use the FACE-Q in research or clinical practice, they are directed to www.qportfolio.org to obtain a license permission from the copyright holders. Reproduction of the FACE-Q in publications without prior permission is not permitted.
